# Memory-driven capture occurs for individual features of an object

**DOI:** 10.1038/s41598-020-76431-5

**Published:** 2020-11-11

**Authors:** Edyta Sasin, Daryl Fougnie

**Affiliations:** Department of Psychology, New York University of Abu Dhabi, Abu Dhabi, United Arab Emirates

**Keywords:** Psychology, Human behaviour

## Abstract

Items held in working memory (WM) capture attention (memory-driven capture). People can selectively prioritize specific object features in WM. Here, we examined whether feature-specific prioritization within WM modulates memory-driven capture. In Experiment 1, after remembering the color and orientation of a triangle, participants were instructed, via retro-cue, whether the color, the orientation, or both features were relevant. To measure capture, we asked participants to execute a subsequent search task, and we compared performance in displays that did and did not contain the memory-matching feature. Color attracted attention only when it was relevant. No capture by orientation was found. In Experiment 2, we presented the retro-cue at one of the four locations of the search display to direct attention to specific objects. We found capture by color and this capture was larger when it was indicated as relevant. Crucially, orientation also attracted attention, but only when it was relevant. These findings provide evidence for reciprocal interaction between internal prioritization and external attention on the features level. Specifically, internal feature-specific prioritization modulates memory-driven capture but this capture also depends on the salience of the features.

## Introduction

The visual world is complex with abundant information. We must select which information is relevant for current processing and for online storage. During perception, selective attention has been shown to improve detection, discrimination and processing of selected sensory input^1–5^. This selective attention mechanism is quite flexible and multifaceted. In addition to being able to direct attention to particular objects, it can also be directed to particular features of the objects—feature-based attention (FBA). For example, we can direct attention to objects with a particular color while ignoring similar objects that have different colors^6–8^. FBA improves behavioral performance and enhances brain response related to task-relevant features while suppressing the non-relevant features^1,9,10^.

Selection also can operate on information stored in the mind. For example, we can select objects to remove from working memory (WM) or objects to prioritize above others based on which objects are more important or more likely to be tested^11–20^. Similar to perceptual attention, selection in the mind can act both on objects or features. Specifically, recent studies demonstrated that retro-cuing specific feature of the stored objects improves memory for the cued feature dimension, at the cost of the uncued feature dimension^21–23^. These findings suggest that WM representations of visual objects can be unbound into separate features and that focusing attention on task-relevant features leads to feature-specific benefits in memory.

That selection can operate on either objects or features is one of many similarities between perceptual attention and selection in the mind (internal attention)^24–28^. Indeed, these two forms of selection have been shown to dynamically interact with each other^29–31^. A good example of such interaction is memory-driven capture—when participants are searching a display while holding other information in memory, distractors that match the contents of memory automatically capture visual attention and disrupt search^32–35^. Importantly, it has also been shown that such automatic capture is reduced or even abolished when an internal representation is retro-cued as no longer relevant^36–38^. These findings suggest that we can prioritize information within WM, and such prioritization can modulate the interaction between perceptual attention and internal selection.

Although the interaction between internal selection and external attention has been studied on the objects level, surprisingly, such interaction has not been investigated on the level of features. This study aimed to fill this gap and test whether prioritizing specific features of the object held in WM will modulate memory-driven capture for objects matching the modulated feature. That is, whether no longer relevant features of an earlier memorized object will still attract visual attention? Given the recent findings showing that retro-cued features are remembered better, while the uncued features are more likely to be forgotten^21–23^, it is reasonable to expect that likelihood of the attentional capture by no longer relevant feature will be reduced. Such a finding would demonstrate that attention and memory can interact at the feature level.

Here participants memorized the color and orientation of an isosceles triangle, and next, they were instructed via retro-cue to remember either the color, orientation, or both features of this triangle. Subsequently, they were asked to execute a search task. To measure attentional capture, we compared search performance on trials when one of the distractors in the display matched the retro-cued or uncued feature (either color or orientation) versus neutral trials when none of the distractors had the same feature value as the previously memorized triangle. We predicted that no-longer relevant (uncued) features would not attract or attract significantly less attention than objects with features that were retro-cued as relevant.

## Experiment 1

### Method

#### Participants

Eighteen students of the New York University of Abu Dhabi (18 females; M = 20.9 years; SD = 2.28) participated in the experiment in exchange for course credit or they were compensated 50 AED per h. We chose a sample size of eighteen, the same sample size used by Sasin et al.^37^ in a similar paradigm investigating the interaction between retrospective attention and attentional capture. All participants had normal or corrected to normal visual acuity and gave informed consent. The experiments were approved by the New York University Abu Dhabi Institutional Review Board in accordance with the ethical principles of the Belmont Report.

#### Apparatus and stimuli

**Figure 1:**
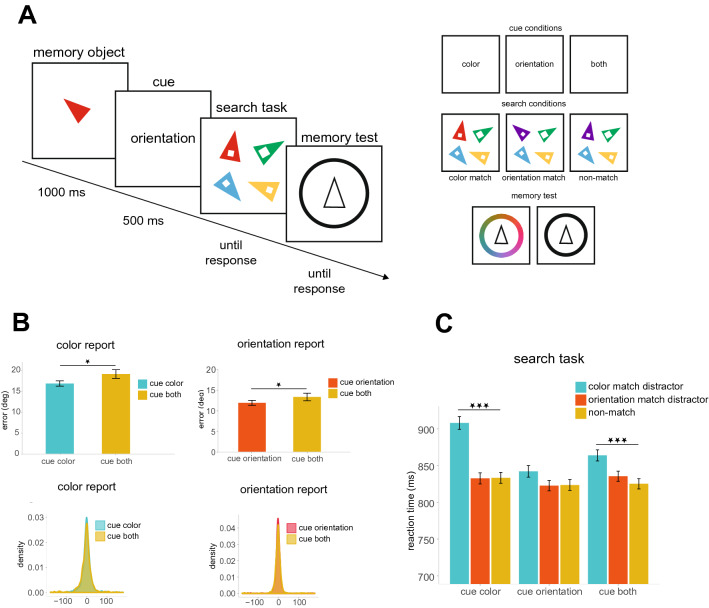
(**A**) Task structure. Participants first remember the color and orientation of a triangle until a retro-cue (100% validity) informs participants to keep in memory either *color*, *orientation,* or *both* features. A subsequent search task asks participants to report the location of the triangle with the largest gap. The color and orientation of the search items are irrelevant for the search and memory task. On *color match* or *orientation match* trials, one of the distractors shared a feature with the memory item. For *non-match* trials none of the search items shared a feature with the memory item. Following the search response, participants were asked to report either orientation or color of the memory item. On cue both trials, color or orientation was randomly selected as the report feature. (**B**) Memory data—Mean absolute error (°, top) and error histograms (bottom) when the reported feature was color (left) or orientation (right). (**C**) Search data—Mean RTs (ms) in the search task as a function of cue condition and match condition. Stars indicate the significance of *t*-test results, *p < 0.05, **p < 0.01, ***p < 0.001. Error bars reflect within-subject standard errors of the mean^40,41^.

Stimuli were constructed and presented using Psychtoolbox for Matlab^39^ and the experiments were run on computers that were fitted with 22-inch BenQ XL2411 monitor (144 Hz refresh rate, 1920 × 1080 pixels). All stimuli were presented on a gray background at a viewing distance of 57 cm. The stimuli used in both the memory and the search task were isosceles triangles. Each triangle had angles of 30°, 75°, and 75° and sides of 1.46°, 2.84° and 2.84° (visual angle) in length. The memory object was a triangle presented centrally at the beginning of each trial. The orientation of the memory object was randomly selected from a set of 180 orientations (2°–360°, in 2° steps). The color of the memory object was randomly selected from a set of 180 equiluminant colors evenly distributed along a circle in the CIE L*a*b* color space (centered at L = 54, a = 18, b =  − 8, with a radius of 59). The word retro-cue could be either: color, orientation, or both. The cue was presented in black Arial font, font size 1.17° of visual angle. The search display consisted of four triangles located equidistantly on an imaginary circle of radius 3.50° around fixation (4.54° of visual angle separated the centers of each item). The triangles in the search display differed by at least 36° in color and orientation. Each triangle had a square gap (Fig. 1A) inside. The target of the search task was designated as the triangle with a large gap (length of 0.66°). The three distractor triangles in the search display had a square gap with a side length of 0.33°.

For the continuous report task, a response wheel with a radius of 3.51° and thickness of 0.44°, appeared around a triangle (with sides of 1.46°, 2.84° and 2.84°, the same size as the triangle in the search task) in the center of the screen. A black indicator bar was displayed outside of the wheel to highlight the currently selected value, which was determined by the angular position of the cursor. For the color probes, the color wheel was composed of 180 colored segments corresponding to the possible stimulus colors. The color wheel was rotated randomly across trials to prevent participants from associating colors at particular angular positions. In the case of orientation probes, the response wheel was black.

#### Procedure

Figure 1A illustrates the procedure and different conditions of Experiment 1. Each trial began with the presentation of a memory object for 1000 ms Participants were asked to remember both the color and the orientation of this object. After a 500 ms blank interval, a retro-cue was displayed for 500 ms, indicating whether the *color*, the *orientation,* or *both* features had to be remembered for a later continuous report (this cue was 100% predictive). Following a blank delay of 1000 ms, the search display was presented until the response was made. There were three search task conditions. On *color match* trials, one of the distractors had the same color as the memory object. On *orientation match* trials, one of the distractors had the same orientation as the memory object. On *non-match trials*, neither of the features of the memory object was shared by any of the objects in the search display. Participants made a speeded mouse press to localize the triangle with the biggest square gap. Participants were informed that the response would be counted as correct if it was within the correct quadrant. The correct quadrant for each of the four triangles was the quarter of the screen on which that triangle was displayed. Mouse position was assigned at the center of the screen at the start of responses. Responses were not registered until participants moved the mouse at least one pixel towards a quadrant, made a click, and released the mouse button. The participant was allowed to select anywhere in the quadrant to select that location.

After a 200 ms blank delay, the memory response screen appeared, asking participants to adjust the color OR orientation of the memory item. The to be reported feature always matched the cued feature. On *cue both* trials, a random report feature was selected. Participants used the mouse to select a particular value (among 180 possible color or orientation values) along a circular response wheel. The color or the orientation (depending on the feature to-be-reported) of a triangle inside the response wheel changed to match the selected value so that participants could match a stimulus to their internal representation. Once participants selected the desired value, they clicked the mouse to give a response. Memory responses were unspeeded, and feedback was provided.

There were 44 trials for each condition, resulting in a total of 396 trials, which were presented in blocks of 33 trials each. Before the full experiment, participants completed nine practice trials.

### Results

#### Memory performance

Memory performance is illustrated in Fig. 1B. Of interest is the absolute deviation in the distance (in degrees of feature space) between the memory report and true value (memory error). We separately analyzed color and orientation errors. Color memory errors were subjected to a 2 (Cue: *color* or *both*) × 3 (Match: *color match*, *orientation match* or *non-match*) repeated measures analysis of variance (ANOVA). The results revealed a main effect of cue, *F*(1, 17) = 6.99, *p* = 0.017, $\eta_{p}^{2}$ = 0.29, showing lower errors in the cue color condition (M = 17.33) than in the cue both condition (M = 19.82). This indicates that the cue successfully improved memory performance. There was no evidence of the search task impacting memory performance. The effect of Match was not significant, *F*(2, 34) = 1.06, *p* = 0.357, $\eta_{p}^{2}$ = 0.06, nor was there interaction between Cue and Match, *F*(2, 34) = 1.32, *p* = 0.280, $\eta_{p}^{2}$ = 0.07.

Orientation memory errors were subjected to a 2 (Cue: *orientation* or *both*) × 3 (Match: *color*, *orientation* or *non-match*) repeated-measures ANOVA. The results matched that found for color. There was a significant effect of cue, *F*(1, 17) = 5.90, *p* = 0.027, $\eta_{p}^{2}$ = 0.26, showing lower memory errors in the cue orientation condition (M = 12.44) than in the cue both condition (M = 14.00). In conjunction with the results for color, this demonstrates better memory performance for the retro-cued feature. The Match conditions had no impact on subsequent memory performance. The effect of Match was not significant, *F*(2, 34) = 2.04, *p* = 0.146, $\eta_{p}^{2}$ = 0.11, nor was there interaction between Cue and Match, *F*(2, 34) = 0.15, *p* = 0.860, $\eta_{p}^{2}$ = 0.01.

#### Search performance

Search reaction times are illustrated in Fig. 1C. Before analyzing the reaction times (RTs) for the search task, we excluded trials with incorrect search responses. Search performance was high, between 97 and 98% for different experimental conditions. Next, we removed outliers in two steps. First, we excluded very short and very long responses (shorter than 150 ms or longer than 3000 ms)^36^. Second, we excluded trials with search-RTs above a cut off value of 3 SD from the mean^42^. This procedure led to removal of 2.38% of the data points. Importantly, the exclusion of outlier trials did not change the qualitative conclusions.

We performed repeated-measures ANOVA on search RTs with Cue (Cue: *color* or o*rientation* or *both*) and Match (Match: *color*, *orientation*, *non-match*) as factors. There was a significant effect of Cue, *F*(2, 34) = 8.52, *p* < 0.001, $\eta_{p}^{2}$ = 0.33. The effect of Match was also significant, *F*(2, 34) = 17.11, *p* < 0.001, $\eta_{p}^{2}$ = 0.50. Critically, we found a significant interaction between Cue and Match, *F*(2, 34) = 2.93, *p* = 0.027, $\eta_{p}^{2}$ = 0.15. To examine this interaction, we compared pairs of different match and non-match conditions across two cue conditions (planned comparisons). In the cue color condition, the RTs were significantly slower on color match trials (M = 908 ms) compared to non-match trials (M = 833 ms), *t*(17) = 4.32, *p* < 0.001, *d* = 1.02. Similarly, in the cue both condition, slower RTs were found on color match trials (M = 864 ms) relative to non-match trials (M = 825 ms), *t*(17) = 3.97, *p* =  < 0.001, *d* = 0.94. Critically, we found that there was no significant difference between color match trials (M = 842 ms) and non-match trials (M = 824 ms), in the cue orientation condition, *t*(17) = 1.29, *p* = 0.215, *d* = 0.30. This suggests that color matching distractors captured attention *only* when color remained task relevant after presentation of the retro-cue. To compare the amount of capture by color for the cue color condition versus cue both retro-cue conditions, we compared attentional capture scores (color match RT–non-match RT). The difference between capture by color in the cue color condition (75 ms) and cue both condition (39 ms) was marginal, *t*(17) = 1.90, *p* = 0.075, *d* = 0.45. Further, while there were significant capture effects from color, we did not observe a difference between orientation match and no-match conditions in either the cue orientation (M = 842 ms vs. M = 823 ms, respectively), *t*(17) = 0.08, *p* = 0.935, *d* = 0.02 or cue both, *t*(17) = 0.95, *p* = 0.358, *d* = 0.22, or cue color conditions *t*(17) = 0.05, *p* = 0.960, *d* = 0.01, suggesting that there was no capture from orientation information at all. Given the lack of capture by orientation, there is no reason to ask if capture by orientation was modulated by the cue.

### Discussion

To sum up, retro-cuing either the color or orientation as relevant led to better memory for respective features compared to when both features had to be maintained in memory. These results are in line with recent studies showing that internal attention can operate on the features level by enhancing and/or protecting representation of the relevant feature dimension^9,21–23^. Moreover, we add to this work by showing that retro-dimension-cue benefits also occur when only a single object is held in WM. Second, we found evidence of memory-driven capture for color. This was reflected by slower search responses when the memory-matching color was present in the search display compared to when none of the colors in the search display matched the item in memory. Finally, and most importantly, we found that memory-driven capture was modulated by the retro-cue. Specifically, attentional capture by color was eliminated when the color was cued as no longer relevant. These results provide first direct evidence for dynamic interaction between internal and external attention on the features level. These findings are also in line with the idea that representations in WM can be decomposed into individual features^23,43–45^. Moreover, our findings suggest a link between attention and working memory at the level of the feature (not just the object), in contrast to the object-based selection hypothesis^46–50^. Results for orientation differed from that for color. We found no evidence of orientation capture in any condition. The lack of capture by orientation could be due to color being a more salient feature compared to orientation. Specifically, while the matching color may more easily stand-out among non-matching colors due to top-down salience attached to this color, the matching orientation might not be as distinguishable among non-matching orientations. This is consistent with stronger guidance for features that are more distinct^51–54^.

## Experiment 2

In Experiment 1, we found no evidence of capture by orientation. One possible explanation for this is that oriented triangle cannot capture attention because it is difficult to extract orientation information when attention is widely spread across the display (in contrast to color which can be perceived and can capture attention regardless of whether the attention window is wide or narrow). To explore this in the next study, we manipulated the locus of the breadth of attention. The hypothesis is that orientation might capture attention when attention is already focused at the location of the matching stimulus before the search presentation. In this case, the matching orientation will be perceived, and participants may be slower to disengage attention. Specifically, the retro-cue (the letter C for color and letter O for orientation) was presented at one of the four possible locations in the visual search display. Crucially, on some trials, retro-cue was presented at the location of memory-matching distractors, and on other trials, it was presented at the location of non-matching distractors. We also included trials on which the retro-cue was presented at the location of the target to ensure participants do not adopt a strategy of shifting attention away from the location of the retro-cue.

### Method

#### Participants

Eighteen students of the New York University of Abu Dhabi (14 females; M = 22.8 years; SD = 2.31) participated in the experiment in exchange for course credit or they were compensated 50 AED per h. All participants had normal or corrected to normal visual acuity and gave informed consent. The experiments were approved by the New York University Abu Dhabi Institutional Review Board in accordance with the ethical principles of the Belmont Report.

#### Apparatus and stimuli

**Figure 2:**
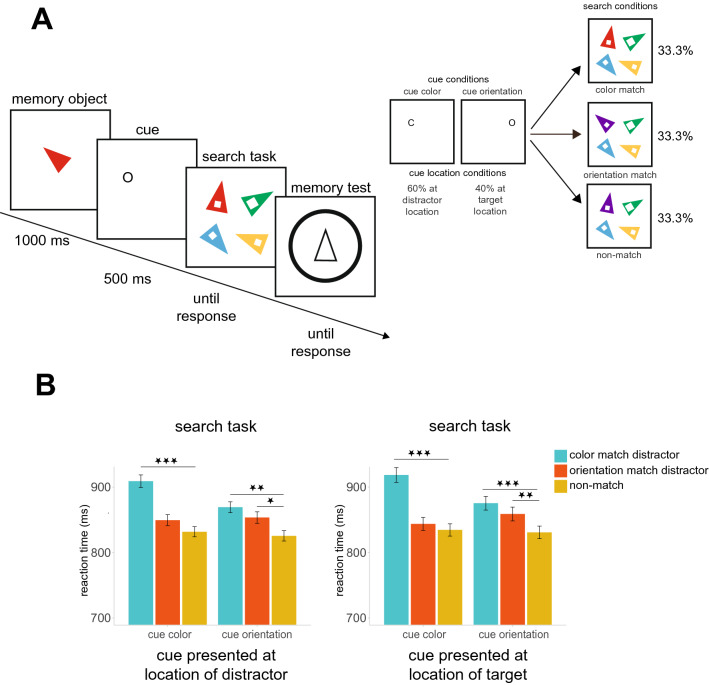
(**A**) Task structure. The method is similar to Experiment 1, except that the cue was now a single letter (C for *color* or O for *orientation*) that was presented either at a target location (40%), or a distractor location (60%). In matching conditions, when the cue was at a distractor location this was always the matching distractor. (**B**) Search data—Mean RTs (ms) in the search task as a function of cue condition and match condition.

The stimuli and apparatus were identical to those used in Experiment 1 except the following changes. The retro-cue indicated whether either color or orientation must be remembered. These retro-cues were in the form of a single black letter “C” and “O”, respectively, presented in Arial font, font size 0.88° of visual angle. Note that the cues were made to be small enough such that differentiating the cues would require participants to focus their attention at the cued location. The cue both condition was not included in Experiment 2 for two reasons. First, the results of Experiment 1, combined with previous results^23^, have clearly demonstrated that retro-dimension-cue can influence behavior. Second, due to the additional conditions related to cue location, it was beneficial to remove the cue both condition to keep the number of trials per condition high. On 40% percent of trials, the retro-cue was presented at the *target location (equally often in all cue and search conditions)*. On 60% of trials, the retro-cue was presented at the *distractor location.* Here, on color or orientation matching trials, the retro-cue was 100% of the time presented at the location of a matching distractor. On matching trials, the retro-cue was never presented at the location of a non-matching distractor as this was not critical to the experiment and would have decreased the trial counts for the critical comparisons. Note that, for non-matching trials (when no matching distractor was shown), the retro-cue could not be presented at the location of a matching distractor (since none was presented). Therefore, in this condition, it was at a random location of one of the non-matching items. The last thing about this design that should be clarified is that we had a slightly bigger number of trials with the retro-cue presented at the distractor location (60%, which were the main subject of interest) than the target location (40%). This difference was small (only 78 trials total) and we do not expect that this impacted the results. Figure 2A illustrates the different types of trials used in Experiment 2.

#### Procedure

The procedure was identical to Experiment 1 aside from the manipulation of cue location.

### Results

#### Memory performance

The effectiveness of the retro-cue was not measured in Experiment 2 as we excluded the cue both condition (and thus, we could not compare memory performance when one feature versus both features were cued). Importantly, while we did not have the full conditions to test for the effect of retro-dimension-cue, we assume that the retro-cue benefited performance, consistent with Experiment 1 and previous findings^21–23^. Memory performance as a function of remaining cue conditions and match conditions is not the main subject of interest here, and they are fully reported in the “Supplemental material”. However, it is worth noting that no significant differences were observed between cue and match conditions.

#### Search accuracy

Search reaction times are illustrated in Fig. 2B. Participant’s performance on the search task was 99% (between 99 and 100% for different experimental conditions). The trial exclusion was handled as in Experiment 1. The incorrect search responses and responses that were shorter than 150 ms or longer than 3000 ms were excluded from RTs analysis. We also removed trials with search-RTs above a cut off value of 3 SD from the mean.

#### Search times: cue location effects

Did the location of the cue impact search performance? To test this, we first conducted an ANOVA on search RTs with the conditions of 2 (Cue Location: *target* or *distractor*) × 2 (Cue: *color* or *orientation*) × 3 (Match: *color match*, *orientation match* or *non-match*). We found significant effect of Match, *F*(2, 34) = 39.37, *p* < 0.001, $\eta_{p}^{2}$ = 0.70. There was also significant interaction between Cue and Match, *F*(2, 34) = 6.95, *p* = 0.003, $\eta_{p}^{2}$ = 0.29. Remaining effects and interactions were not significant (all *F*’ < 2.98, all *p*’s > 0.101). Thus, in terms of the impact on search performance, there was no evidence that cue location impacted search. Thus, for all remaining analyses, the data were collapsed across different cue locations. Importantly, equivalent qualitative results were found if separate analyses were performed on the data where the cue was at the target versus a distractor location (see “SupplSupplemental material”).

The lack of differential effects of capture when the cue was at a target versus a distractor location is surprising. However, we suspect that this occurs because our task involves a comparative judgement (finding the biggest gap) and thus forces participants to check multiple search items. Thus, even when the cue was at a target location, participants are likely to check an item with a matching distractor. In conjunction with the results of Experiment 1, the findings suggest that it is more about *whether* attention is focused and less about *where* attention is focused.

#### Search times: capture effects

Because the location did not modulate the capture effects, next, we tested capture effects (i.e., the search performance difference between matching and non-matching trials) on data that were collapsed across cue location conditions. To examine influence of Cue and Match on search performance a 2 (Cue: color or orientation) × 3 (Match: color match, orientation match or non-match) repeated measures ANOVA was performed on mean RTs. The effect of Match, *F*(2, 34) = 39.38, *p* < 0.001, $\eta_{p}^{2}$ = 0.70, and interaction between Cue and Match, *F*(2, 34) = 6.94, *p* = 0.003, $\eta_{p}^{2}$ = 0.29 were significant. The effect of Cue was not significant, *F*(1, 17) = 2.99, *p* = 0.102, $\eta_{p}^{2}$ = 0.15.

To examine the interaction between Cue and Match, we compared pairs of different Match conditions across two Cue conditions. First, we examined the attentional capture by color. Planned comparison showed slower RTs on the color match trials (M = 914 ms) versus non-match trials (M = 833 ms) in the cue color condition, *t*(17) = 7.25, *p* < 0.001, *d* = 1.71. Slower RTs on color match trials (M = 856 ms) compared to non-match trials (M = 828 ms) were also found in the cue orientation condition, *t*(17) = 4.46, *p* < 0.001, *d* = 1.05. Thus there was evidence for attentional capture for color both when color was cued and when orientation was cued. However, the amount of capture (color match RT–color non-match RT) was larger when color was cued (M = 81 ms) than when orientation was cued (M = 44 ms), indicating that color being task-relevant implies greater capture from color, *t*(17) = 2.93, *p* = 0.009, *d* = 0.69.

Second, we examined the attentional capture by orientation. We found slower RTs on orientation match trials (M = 856 ms) compared to non-match trials (M = 828 ms), *t*(17) = 4.46, *p* < 0.001, *d* = 1.05, in the cue orientation condition. The RTs did not differ between orientation match trials (M = 847 ms) and non-match trials (M = 833 ms) in the cue color condition, *t*(17) = 1.15, *p* = 0.266, *d* = 0.27, suggesting that orientation capture is only observed when orientation is task-relevant. However, the captures by orientation (orientation match RT–orientation non-match RT) in the cue orientation condition (M = 28 ms) and cue color condition (M = 13 ms) did not significantly differ from each other, *t*(17) = 1.10, *p* = 0.287, *d* = 0.26. Thus, while we have evidence that orientation can capture attention in this study, the evidence is not clear on whether orientation capture is modulated by the cue.

#### Discussion

To sum up, we found attentional capture when either color or orientation of memorized object was cued as task-relevant. When a particular feature was uncued and thus no longer relevant, the attentional capture by this feature was significantly reduced (color) or not present (orientation). Although the analysis showed no evidence of capture by orientation when orientation was irrelevant, the difference in the amount of capture when orientation was relevant versus when it was irrelevant did not reach significance level. It is likely that capture effects by orientation, which were quite small, would require more power to observe clear modulation of these captures by retro-cue. Taken together, these findings suggest that internal selection induced by the retro-cue not only modulates the content of WM but also modulated how external attention is deployed. Some aspects of the current findings require a closer look. First, we found attentional capture by orientation, which was not observed in Experiment 1, mostly when orientation was task-relevant. The notable change was that in Experiment 1, the cue was presented centrally, whereas in Experiment 2, the cue directed attention to a specific location in the search display. Surprisingly, the capture by orientation occurred both when attention was pre-focused on the location of upcoming distractors or targets. Presenting the letter cue at a specific location may lead participants to narrowly pre-focus on this specific location (perhaps even accompanied by an overt eye movement), which in turn may lead to more elaborate processing of the distractor subsequently occurring at this location. In contrast, in Experiment 1, centrally presented word cue attracted attention to the center, in which case memory-matching orientation was less likely to be extracted from the search display. Indeed, rather than reflecting a property intrinsic to color or orientation per se, our results may reflect the fact that orientation information may not have been efficiently extracted from our stimuli without either focused covert or overt attention (i.e. eye movements) on a particular stimulus.

The fact that capture occurred even when the cue was at the matching location suggests that one underlying mechanism might be a slower disengagement of attention when a distractor matches task-relevant features with an item in memory^36,55^. Moreover, because we used a task that required participants to detect the triangle with the biggest square gap, this may have induced participants to engage in a comparative task. Even when the target appeared at the attended location, participants might need to compare the gaps on multiple triangles to identify the one with the large gap.

## General discussion

In the laboratory, researchers typically use working memory tasks where the to-be-remembered information does not change during the trial. However, in the real world, the contents of our memory are dynamic. In particular, the feature contents or the relevant features could change over time. For example, while shopping, you may want to change from looking for a t-shirt of a particular color to looking for a shirt with stripes. What are the implications of this for memory-driven capture? Does visual input matching *feature* information that was encoded into WM but is no longer relevant still grab external attention? (see^36,37,56–58^ for evidence of this at the *object* level). To answer this question, we asked people to memorize the color and orientation of an isosceles triangle. Next, we used a retro-cue to indicate whether either the color, the orientation, or both features of this triangle are relevant for a later continuous report. Subsequently, participants search for a target among distractors. We measured memory-driven capture by comparing whether search performance was impaired when there was a distractor matching one of the features of the previously memorized object. Experiment 1 showed that retro-cuing specific features led to better memory for this feature compare to when both features had to be held in WM. These findings suggest that internal prioritization can selectively enhance activation of the specific feature of the object held in WM^21–23^. Crucially, the results of Experiment 1 showed that memory-driven capture was modulated by the retro-cue—capture disappeared for color when it was cued as no longer relevant. This finding shows that shifting attention to a specific feature in WM has an impact on attention. Because in Experiment 1, we found no capture by orientation (regardless of its relevance), in Experiment 2, we focus participants’ attention during the cue stage, before appearance of the search array. We observed some modulation of attentional capture by the retro-cue for both color and orientation in Experiment 2. However, evidence for retro-cue modulation of capture by orientation was weak. This is the first study showing that internal feature specific modulation within objects leads to feature specific modulation of memory-driven capture.

What is the mechanism underlying this modulation of memory-driven capture? Previous literature has proposed several mechanisms that could explain the benefits of valid retro-cues and the costs of invalid retro-cues^18^. According to one hypothesis, the no longer relevant information is not protected from perceptual interference, and thus this information is more likely to be forgotten^14,59,60^. Another hypothesis claims that the benefit comes from removing the no longer relevant information as this frees limited resources for other items^19,61–64^. Lastly, the time-based decay hypothesis assumes that WM representations deteriorate over time and that internal prioritization aid in counteracting this process^17^. Future research is required to understand the exact mechanism(s) that are contributing to the retro-cue modulation of memory-driven attentional capture observed in the current study.

While we found clear benefits of the retro-cue on memory performance, and modulation of memory-driven capture, it is important to remark that the lack of capture by the no longer relevant feature does not necessarily mean that this feature was completely removed from WM. Indeed, there is neuroimaging evidence that uncued information is not completely lost from memory and that the information can be reconstructed by measuring brain response to impulse stimulus presented during maintenance interval^65^ or by measuring brain response after targeted a pulse of transcranial magnetic stimulation^66^. One possibility is that cued versus uncued information are in different states of memory with the memories associated with the cued representation being closer linked to attention (active state). The uncued or unattended information might be still held in a more latent state (in case it is relevant later)^65–69^.

The current study shows that features are not necessarily equal in regards to the memory-driven capture effect. Specifically, we found less capture for orientation than color (particularly in Experiment 1 in which no capture by orientation was found). The lack of capture by visual input matching orientation information held in WM may be surprising, especially in light of the visual search studies suggesting that orientation is one of the “preattentive” features that can efficiently guide attention during visual search^8,70–73^. However, these studies are focused on goal-directed guidance of attention. At the same time, memory-driven capture is based on guidance that is irrespective of the goals of the observer, and thus these two situations are quite different. Although orientation has been shown to be effective in top-down guidance in search, there is evidence in the literature showing that items defined by color are detected faster than items define by orientation^74,75^. Further, our orientation information was the direction of an isosceles triangle, and this type of information may be less distinguishable from each other than e.g. different colors, and therefore, less likely stand out among other non-matching orientations. However, orientation information might be more likely to be extracted if it appears on the location when attention is focused (either covertly or overtly). Consistent with this, when attention was narrowly focused prior to search display onset in Experiment 2, we observed capture by orientation, regardless of whether this was a matching or non-matching location. Our task required a comparative decision about the property of the items, which may have led participants to compare multiple items even when the matching location was attended first. One interpretation of this capture is that it is from slower disengagement of attention from memory-matching orientations than from non-matching orientations.

Taken together, the current study reinforces the notion that attention and memory are strongly interconnected processes. Previous research provided extensive evidence showing that information held in WM influences the allocation of visual attention^31,33,35,47,76,77^. Such influence has also been shown to be sensitive to change in the relevance of the information in WM. Our work adds to the literature on this topic by demonstrating that the dynamic interaction between prioritization in memory and attentional capture occurs at the level of features, not just objects.

## Supplementary information

Supplementary Information.

## Data Availability

The data for all experiments can be accessed in the Open Science Framework (https://osf.io/faecw/).
