# Supplementary material for: Memory-driven capture occurs for individual features of an object
Source: Sci Rep. 2020 Nov 11;10:19499. doi: 10.1038/s41598-020-76431-5 (PMC7658969; doi:10.1038/s41598-020-76431-5)
Supplement: Supplementary file 1 — Supplementary Information. [file 41598_2020_76431_MOESM1_ESM.docx]

Memory-driven capture occurs for individual features of an object.

Edyta Sasin and Daryl Fougnie

**Supplemental Material**

**Memory performance in Experiment 2** (Figure 1)**.** To examine memory performance, first, we conducted a repeated-measures ANOVA with the Match as a factor (Match: *color match*, *orientation match* or *non-match*) on color reproduction errors separately for trials with cue presented at the distractor location and cue presented at the target location. There were no significant effects, neither interaction (all *F*’s < 0.61, all *p*’s > .515) for color errors.

A repeated-measures ANOVA with Match as a factor (Match: *color match*, *orientation match* or non-match) on orientation reproduction errors separately for trials with cue presented at the distractor location and cue presented at the target location also revealed no significant effects (all *F*’s < 1.31, all *p*’s > .285) for orientation errors.

**Fig.1** Memory data—Mean absolute error (°) as a function of Match. Error bars reflect within-subject standard errors of the mean.

**Capture effects in Experiment 2:**

*Capture effects: Cue at DISTRACTOR locations*: To examine the influence of the cue on search performance when the cue was at a distractor location, a 2 (Cue: *color* or *orientation*) × 3 (Match: *color match*, *orientation match* or *non-match*) repeated measures ANOVA was performed on mean RTs for trials when cue appeared at the location of the distractor. The effect of Match, *F*(2, 34) = 16.27, *p* < .001, $\eta_{p}^{2}$= .49, and interaction between Cue and Match, *F*(2, 34) = 4.79, *p* =.015, $\eta_{p}^{2}$= .22, were significant. The effect of Cue was not significant, *F*(1, 17) = 1.86, *p* =.190, $\eta_{p}^{2}$= .09.

To examine the interaction between Cue and Match, we compared pairs of different Match conditions across two Cue conditions. First, we examined the attentional capture by color. Planned comparison showed slower RTs on the color match trials (M = 909 ms) versus non-match trials (M = 832 ms) in the cue color condition, *t*(17) = 5.26, *p* < .001, *d* = 1.24. Slower RTs on color match trials (M = 869 ms) compared to non-match trials (M = 825 ms) were also found in the cue orientation condition, *t*(17) = 3.52, *p* = .003, *d* = 0.83. Thus there was evidence for attentional capture for color both when color was cued and when orientation was cued. However, the amount of capture (color match RT – color non-match RT) was larger when color was cued (M = 77 ms) than when orientation was cued (M = 44 ms), indicating that color being task-relevant implies greater capture from color, *t*(17) = 2.85, *p* = .011, *d* = 0.67.

Second, we examined the attentional capture by orientation. We found slower RTs on orientation match trials (M = 853 ms) compared to non-match trials (M = 825 ms), *t*(17) = 2.20, *p* = .042, *d* = 0.52, in the cue orientation condition. The RTs did not differ between orientation match trials (M = 849 ms) and non-match trials (M= 832 ms) in the cue color condition, *t*(17) = 1.27, *p* = .220, *d* = 0.30, suggesting that orientation capture is only observed when orientation is task-relevant. However, the difference between capture by orientation in the cue color (M = 18 ms) versus cue orientation condition (M = 28 ms) was not significant, *t*(17) = 0.68, *p* = .506, *d* = 0.16.

*Capture effects: Cue at TARGET locations:* We performed a 2 (Cue*: color* or *orientation*) × 3 (Match: *color match*, *orientation match* or *non-match*) repeated measures ANOVA on mean RTs for trials when cue appeared at the location of the target. Again, the effect of Match, *F*(2, 34) = 31.88, *p* < .001, $\eta_{p}^{2}$= .65, and interaction between Cue and Match, *F*(2, 34) = 4.94, *p* =.013, $\eta_{p}^{2}$= .23, were significant. The effect of Cue was not significant, *F*(1, 17) = 2.48, *p* =.133, $\eta_{p}^{2}$= .13. To examine the double interaction, we compared pairs of different match and non-match conditions across two cue conditions.

First, we examined the attentional capture by color. RTs were slower on color match trials (M = 918 ms) vs. non-match trials (M = 835 ms), in the cue color condition, *t*(17) = 7.75, *p* = .<001, *d* = 1.83. The slower RTs on color match trials (M =875 ms) versus non-match trials (M = 831 ms) were also observed in the cue orientation condition, *t*(17) = 4.35, *p* = .<001, *d* = 1.02. Retro-cuing the color led to larger capture effect (color match RT – non-match RT) than retro-cuing the orientation (M = 84 ms vs. M = 45 ms, respectively),*t*(17) = 2.21, *p* = .041, *d* = 0.52. These findings again demonstrate that the magnitude of capture by matching color is reduced when color is retro-cued as task-irrelevant.

Second, we examined the attentional capture by orientation. The slower RTs were observed on orientation match trials (M = 859 ms) compared to non-match trials (M = 831 ms), in the cue orientation condition, *t*(17) = 3.09, *p* = .007, *d* = 0.73. There was no difference in RTs between orientation match trials (M = 844 ms) and non-match trials (M = 835 ms), in the cue color condition, *t*(17) = 0.63, *p* = .541, *d* = 0.15. However, again the difference in capture effects between cue color (M = 9 ms) and cue orientation condition (M = 28 ms) was not significant, *t*(17) = 1.01, *p* = .327, *d* = 0.24.
